# Presence of the Reflective and Critical Thinking Capacity in Nursing Curricula in Iberian America

**DOI:** 10.17533/udea.iee.v38n3e14

**Published:** 2020-11-11

**Authors:** Lucila Cárdenas Becerril, María Antonia Jiménez-Gómez, María Dolores Bardallo-Porras, Jesús López-Ortega, Araceli Monroy-Rojas, Vilanice Alves de Araújo-Püschel

**Affiliations:** 1 Nurse, Ph.D. Professor, Universidad Autónoma del Estado de México, México. Email: lucycabe62@yahoo.com Universidad Autónoma del Estado de México Universidad Autónoma del Estado de México Mexico lucycabe62@yahoo.com; 2 Nurse, Master’s. Associate professor, Universidad Nacional de Colombia, Bogotá, Colombia. Email: majimenezd@gmail.com Universidad Nacional de Colombia Universidad Nacional de Colombia Bogotá Colombia majimenezd@gmail.com; 3 Nurse, Ph.D. Professor, Escuela Superior de Enfermería del Mar, Barcelona, España. Email: mbardallo@psmar.cat, redriiee@gmail.com Escuela Superior de Enfermería del Mar Barcelona España mbardallo@psmar.cat redriiee@gmail.com; 4 Nurse, Ph.D. Professor, Universidad de Jaén, España. E-mail: jlortega@ujaen.es Universidad de Jaén Universidad de Jaén Spain jlortega@ujaen.es; 5 Nurse, PhD. Universidad Autónoma Metropolitana Xochimilco, México. Email: aramonroy@yahoo.com Universidad Autónoma Metropolitana Xochimilco México aramonroy@yahoo.com; 6 Nurse Ph.D. Associate professor, Universidade de São Paulo, Brasil. Email: vilanice@usp.br Universidade de São Paulo Universidade de São Paulo Brazil vilanice@usp.br; 7 Red Iberoamericana de Investigación en Educación en Enfermería (RIIEE)

**Keywords:** education, nursing, thinking, curriculum, students, nursing, educación en enfermería, pensamiento, curriculum, estudiantes de enfermería, educação em enfermagem, pensamento, currículo, estudantes de enfermagem

## Abstract

**Objective.:**

The objective was to identify the presence of the capacity for reflexive-critical thinking or similar, in Nursing Curricula in Iberian America.

**Methodology.:**

.The article gathers the results of one of the objectives of the macro-project developed by the Iberian American Network on Nursing Education Research, titled *Strategies to develop reflective and critical thinking in nursing students* **:** *Iberian America situation* **.** To achieve this, a descriptive and exploratory research was conducted with qualitative approach. An instrument created for this project was used, along with some guiding questions to focus the information.

**Results.:**

Eight countries participated (Bolivia, Brazil, Colombia, Ecuador, Spain, Mexico, Peru, and Venezuela), which contributed information from 189 curricular plans. The R&CT was found in the majority of the curricula, although with diverse denominations. The principal learning strategies used were problem-based learning, group dynamics, reflective reading, clinical practice, and simulation laboratories. The evaluation methods used are the knowledge test, case analysis, and practical exam.

**Conclusion.:**

Significant stress exists in the discourse and curricular organization. Incongruences were found and a clear inclination toward the formation of professionals with broad technical skills under a traditional, memory, banking and knowledge accumulation education.

## Resumen

**Objetivo.:**

Identificar la presencia de la competencia de pensamiento reflexivo-crítico o afines en los currículos de Enfermería en Iberoamérica.

**Metodología.:**

El artículo recoge los resultados de uno de los objetivos del macro-proyecto que desarrolla la Red Iberoamericana de Investigación en Educación en Enfermería (RIIEE) titulado *Estrategias para desarrollar en el estudiante de enfermería el pensamiento reflexivo y crítico* (PRyC)**:** *situación Iberoamérica* **.** Para lograrlo se realizó una investigación descriptiva y exploratoria con abordaje cualitativo. Se empleó un instrumento creado para este proyecto y algunas preguntas orientadoras para focalizar la información.

**Resultados.:**

Participaron ocho países (Bolivia, Brasil, Colombia, Ecuador, España. México, Perú y Venezuela) que aportaron la información de 189 planes curriculares. El PRyC se encontró en la mayoría de los currículos, aunque con diversas denominaciones. Las principales estrategias de aprendizaje empleadas fueron: el aprendizaje basado en problemas, las dinámicas grupales, la lectura reflexiva, la práctica clínica y los laboratorios de simulación. Los métodos de evaluación utilizados son el examen de conocimientos, el análisis de casos y el examen práctico.

**Conclusión.:**

Existe una tensión significativa en el discurso y la organización curricular. Se encuentran incongruencias y una clara inclinación hacia la formación de profesionales con amplias capacidades técnicas bajo una educación tradicional, memorística, bancaria y de acúmulo de conocimientos.

## Resumo

**Objetivo.:**

O objetivo foi identificar a presença da competência de pensamento reflexivo e crítico ou relacionado, nos currículos de enfermagem na América Latina.

**Métodos.:**

O artigo reúne os resultados de um dos objetivos do macroprojeto desenvolvido pela Rede Ibero-americana de Pesquisa em Educação em Enfermagem (RIIEE) intitulado Estratégias para Desenvolver o Pensamento Reflexivo e Crítico em Estudantes de Enfermagem (PRyC): a situação ibero-americana. Para conseguir, foi realizada uma pesquisa descritiva e exploratória com abordagem qualitativa. Um instrumento criado para este projeto e algumas perguntas orientadas para enfocar as informações.

**Resultados.:**

Participaram oito países (Bolívia, Brasil, Colômbia, Equador, Espanha, México, Peru e Venezuela) que contribuíram com informações de 189 planos curriculares. O PRyC foi encontrado na maioria dos currículos, embora com nomes diferentes. As principais estratégias de aprendizagem utilizadas foram: aprendizagem baseada em problemas, dinâmica de grupo, leitura reflexiva, prática clínica e laboratórios de simulação. Os métodos de avaliação utilizados são o teste de conhecimentos, a análise de casos e o teste prático.

**Conclusão.:**

Existe uma tensão significativa no discurso e na organização do currículo. Há incongruências e uma clara inclinação para a formação de profissionais com amplas capacidades técnicas sob uma educação tradicional, de memória, bancária e de acumulação de conhecimento.

## Introduction

Currently, the nursing professional’s formation must respond in efficient, integral, timely, assertive, and humanistic manner to the demands required by caring for life and maintenance and reestablishment of human health. This is not an easy task, given that the formal care provided by this professional implies the dynamic of growing changes in the designs and current university methodologies, claim with it the implementation of an integrating vision and transversal of the contents, to deploy effectively the elements necessary to manage to strengthen the skills needed by a graduate from a professional nursing program.

Diverse educational organisms, like UNESCO or the Latin American Association of Faculties and Schools of Nursing (ALADEFE, for the term in Spanish), recommend que las higher education institutions (HEI) in Iberian America que offer higher education in Nursing (undergraduate and graduate), form and contribute an interpretative vision in nursing professionals, through an academic curriculum that goes beyond teaching the diverse functions -granting, education, investigation, management, and administration, among others - that will take place around formal or professional nursing care, developing innovative skills.(1) One of the cross-sectional curricular axes that must be considered is the development, implementation, and promotion of reflective and critical thinking (R&CT), which offer nursing professionals the theoretical-practical tools necessary to use this type of thinking in any environment of the labor market where they are inserted, which will enhance their vision, autonomy, and professional and social recognition**.**(2,3)

Thinking reflectively and critically permits knowing and discriminating one action from another, in function of priorities established to care for the person, interacting with them in emancipatory manner, where forms or bridges of union must be sought between the most artistic part of nursing and those elements more associated with the field of science, thus, achieving higher quality when providing care.(4) The development of R&CT in nursing students in Iberian America has been poorly studied, although it increasingly accepted that this type of thinking must be a skill upon graduation of all the programs to form nursing professionals. The aforementioned is justified by the relation critical thinking has with the nursing staff’s capacity to make clinical judgment in the nursing practice, which is necessary to provide therapeutic care of the highest quality. Additionally, it has been indicated that nursing professors are responsible for the creation and implementation of curricula that form and graduate professionals capable of using the skills of critical thinking.(5)

The critical thinking capacity describes an integral or holistic behavior and it is expected that its promotion and development be cross-sectional, that is, that during each period and in each assignment or unit of learning it is taught by the professor and learnt by the students, as an individual skill, with interpersonal connotations, related with other skills, like analysis and synthesis capacity, critical and self-critical capacity, management of information, decision making, and problem resolution.(6)

Since the 1990s, in different national and international institutional documents, the need was established to develop the capacity for R&CT of the nursing staff during their formation and during exercise of their work practice. The scientific literature related with this study object also confirms the importance of developing said capacity.(7,8) Nevertheless, there is a shortage of investigations related with the nursing discipline about the pedagogic and didactic practice to advance in this way of thinking. Hence, it becomes a priority for the nursing faculty staff to identify the situation that prevails in teaching and learning R&CT as a capacity that must be developed and promoted during the whole formation process of new generations, besides privileging during the professional exercise, in any setting of the nursing labor market.

The Iberian American Network on Nursing Education Research (RIIEE, for the term in Spanish) identified as one of its research priorities the “Development of Reflective and Critical Thinking in Nursing Students”, a preference backed by the participation of over 80 nurses from Iberian American countries who attended the 3^rd^ Network Meeting in September 2011, in Coimbra, Portugal, within the framework of the 11^th^ Iberian American Conference on Nursing Education, organized by the ALADEFE. This article describes the presence of the R&CT capacity in curricula of higher level or degree in nursing of educational institutions in Iberian America, as well as the educational approaches, teaching, learning, and evaluation strategies registered in each academic program analyzed.

More frequently, the characteristics that distinguish nursing professionals are cognitive abilities over psychomotor skills that, historically, have been over-dimensioned in the academic curriculum. Reflection and reflective practice are very familiar terms, currently, within the setting of the nursing practice. These are meanings that can generate a broad spectrum of sensations and reactions: from more enthusiastic adhesion to ambiguity and skepticism. In recent decades, ideas related with reflection and the reflective practice have been incorporated in initial and continuous formation programs in the context in which is conducted the research study by the RIIEE.(9)

The evolution of nursing degree curricula has crossed continental borders and has homogenized the objectives and las philosophical lines that inspire different curricula aimed, in these moments, at formation through skills, among which are highlighted as vertebral axis of the formation the capacity for critical thinking. This has permitted, saving the cultural, economic, and socioeconomic diversity of the different regions, to undertake an interuniversity and international study of the educational dimensions presented by this research.(10) Given that the curriculum stems from the elaboration of school programs and from the conformation of educational systems that should address the need to achieve greater efficiency of educational systems(11) within a given context, it is said to originate from a cultural construction, within a balance between the formation as professional, as person, and as member of a culture, to organize a series of educational practices in the formation of professionals.

This research has considered the paradigmatic visions or positioning of the authors already mentioned, from which was constructed the integrating notion of R&CT by the RIIEE, which alludes to a “Process of complex, systematic, dialogical and deliberate, self-directed and action-oriented reasoning, whose primary purpose is to choose, based on intellectual and affective processes (cognitive, experiential and intuitive), the best response options that favor nursing problem solving in well-defined contexts and according with the ethical postulates of the profession”.(12) This concept guides the study carried out by the RIIEE and incorporates the dialogical and participative attribute of critical thinking, like the expression of the maturity of thought, from the constructivist-critical perspective. Moreover, the cognitive, experiential, emotional, contextual, and ethical dimensions can be identified in the exercise of this way of thinking, capacities present in the epistemology of the nursing practice. To achieve the objectives of the nursing professionals, these need to generate, promote, and apply R&CT in each and all of their actions. It is essential, then, that from their formation, the new generations learn, comprehend, and apprehend this form of reasoning, which will allow a timely, effective, efficient Being, Knowing and Doing with high epistemological and sociological impact.

The objective of this study was to identify the R&CT presence or similar (critical thinking, reflexive thinking, analysis and synthesis, critical and self-critical capacity, information management, decision making, problem resolution, among others), in the different Nursing curricula of higher education institutions in Iberian America.

## Methodology

This research was carried out with the participation from research groups from Bolivia, Brazil, Colombia, Ecuador, Spain, Peru, Mexico, and Venezuela. It is an exploratory, descriptive study with mixed approach. This article privileges the presentation of qualitative results. Although the project has a common methodological framework, consensual adaptations have been made, in function of the context in which it has been applied, according to the exploratory methodology adopted, to obtain results more in keeping with the reality in each region and assuming the characteristics and limitations of each context. Regarding the *study universe,* these were nursing curricula of Iberian America and the *study population* the nursing curricula from universities that offer the career in Nursing in the regions comprising the RIIEE, independent of their being public or private. A population was chosen through convenience, taking into account the complexity and diversity that exists in a study of these dimensions and areas of influence, as shown in Table 1.

**Table 1:** Number of curricula analyzed from the region of Iberian America

Country	Number
Bolivia	7
Brazil	45
Colombia	38
Ecuador	15
Spain	31
Mexico	26
Peru	21
Venezuela	6
Total	189

The inclusion criteria were the curricula that had in their academic plan some assignments that included the R&CT capacity or similar words considered synonymous for this study, such as critical thinking, reflexive thinking, analysis and synthesis, critical and self-critical capacity, information management, decision making, problem resolution, among others; the curricula had to of public access or that when required from the respective academic instances, such would be provided.

The study categories or nuclei were: (i) presence of the R&CT capacity or similar in the curriculum; (ii) teaching and learning methodologies and strategies suggested in the curricula to develop and promote the R&CT capacity or similar; (iii) forms of evaluations suggested in the nursing curricula linked to achieving the R&CT capacity; and (iv) coherence between the R&CT capacity and the educational methodology used in teaching, learning, and assessment processes. Upon identifying the R&CT presence in the curricula, the study proceeded to review the areas of knowledge or areas of development of the academic offer, which were classified into biomed, public health, nursing, research, and electives.

To collect the data, a document was constructed, which in the first instance permitted identifying the type of institution, dependence, and country; thereafter, it was possible to gather elements to characterize R&CT within the curricula, through data related with the presence and denomination of this capacity, where, through consideration of the research team, terms were accepted, like analysis and synthesis, problem resolution, decision making, information management, or critical and self-critical capacity, given that they are all related with this type of thinking. Data was also gathered about the assignments, areas of knowledge, teaching-learning methodologies, and evaluation activities, with these elements being necessary to complement the characterization of the development and positioning of R&CT within the curricula. Lastly, to guide filling out the document, 10 guiding questions were elaborated, which permitted obtaining information on the educational approaches and the curricular theory that support the development of R&CT in nursing education institutions in Iberian America that participated in the research.

The empirical or field phase filled out 189 data-collection documents. The results were first grouped into the four regions that participated in the research: Andean, Brazil, Mexico, and Europe, resulting from it a regional analysis to, finally, elaborate a final report that permitted knowing the differences and similarities in the nursing degree academic programs in eight countries and 189 curricular plans; organized based on the four categories or thematic nuclei of study already indicated, deriving from the results some conclusions, reflections, and recommendations, taking as axis the objectives proposed in this research.

This project adhered to the Helsinki Declaration and to legislation in effect in each region and country of Iberian America for the regulation of confidentiality and protection of data obtained in the research.

## Results

### R&CT presence capacity or similar in the curriculum

The total of the curricula reviewed, although with different emphasis, contemplate the development of R&CT as priority for all graduates from educational institutions. It was identified that this type of thinking was found in three complementary stages: as formation purpose, as methodological strategy, and as result. The *formation purpose* proposes achieving a professional with integral, reflexive, critical formation, capable of adapting and transforming reality, with critical conscience, entrepreneurial leader, free, critical, and universal. The *methodological strategy* develops and implements pedagogical methods that promote reasoning and creative critical thought, curricular design through skills, analysis of lectures, group dynamics, individual and group practices, integrates theory-practice and integrates teaching-research and extension, with respect to conducting research, which can transcend at interdisciplinary and transdisciplinary levels. As *result*: the individual can solve problems, promote critical thinking, generate knowledge, critical analysis, capable of anticipating and visioning the future, and construct viable alternatives to problems.

Referring to the curricular theory used in the construction and conduction of the curricula, the study identified diversity of approaches, although in most cases alluding to constructivism, centered on learning (independent learning or self-learning), through skills, flexible or semi-flexible; aimed in inter and/or multidisciplinary manner, favoring, in some cases, accent lines: community, clinical, entrepreneurial, educational and/or research, although it is worth mentioning that, mostly, the skill focuses only on the critical capacity and skill, leaving aside the reflective element. The explicit and implicit R&CT presence was found in all the elements that make up the curriculum, such as mission, vision, objectives, foundation of the academic program, graduation profile, curricular guidelines and evaluation, among others. Although it may be said that great diversity exists of terms that, based on the sense in which they are employed within the curricula, these may be considered synonyms of R&CT. Thus, the following can be stated: critical knowledge, reflexive thinking, analysis and synthesis, clinical practice, problem solving capacity, critical and self-critical capacity, critical and/or reflective attitude, critical and/or reflective skill, critical and/or reflective capacity, clinical judgment or clinical method, critical spirit, problem resolution, information management and decision making; all imply a self-directed, complex, systematic, and deliberate reasoning process aimed at action.

Within the Latin American RIIEE setting (Andean, Brazilian, and Mexican regions), R&CT presence is mostly consigned to areas of lower curricular weight, like the *Social-humanist*, which included assignments, like sociology, values, ethics, among others, many of them of elective nature; in contrast to those of greater curricular weight, like that of *disciplinary-medical-technical knowledge* - integrated by assignments, like anatomy, physiology, genetics, pharmacology, and nursing (basic, surgical, pediatric, maternal infant, among others). Other assignments in which the capacity appears are Communication skills, Foreign languages, Research, Information management, Workshops, Informatics and Technology. In Spain (European region), this capacity is specified predominantly in areas of disciplinary knowledge, like Nursing models and theories, Nursing process, *Practicum*, Anthropology of Health, and Epistemology of Nursing.

It was common to find in the graduation profile of the Nursing Curricula, belonging to the different institutions studied, the following statements: “train higher-level professionals committed with the population’s health, development of their discipline, with capacity to construct from their own knowledge, take initiative and solve problems; competitive and with aptitude for team work, assuming responsibly the risks involved in disciplinary and interdisciplinary professional practice, capable of applying knowledge based on scientific evidence to care for human health, with humanistic and bioethics sense”; additionally, attributes are expressed on knowledge, skills, attitudes, and values to meet the needs, demands, and conditions of the nursing labor market for the 21^st^ century, making broad specifications on the *Being, Knowing and Doing* of the nursing professional. An example of this is the following manifestation: *“the Nursing degree graduate will be able to …solve problems, followed by decision-making professionals, with critical thinking, creative, participant, enterprising, productive, reflective and self-critical, capable of making clinical judgments, researcher, capable of business creation and engaging in independent practice, with autonomy, creativity, and self-realization, competent for intersectoral, multidisciplinary work, with effective communication and oral and written expression. Capable of providing integral care, conducting continuous and permanent learning, according to personal and professional needs…”* (C-32).

### Teaching and learning methodologies and strategies suggested in the curricula to develop and promote R&CT capacity or similar.

Each curriculum analyzed consigns onto the “teaching and learning activities” section a range of actions the curricular program suggests, but it should be mentioned that this is only the so-called *formal or written curriculum*, which may not necessarily be congruent between the real and the hidden curriculum*,* an aspect that in this study exceeds those comparisons. Hence, this section only accounts for what was found in most of the curricula reviewed. In all the regions studied, the academic programs make explicit the teaching-learning strategies and evaluation of the capacity for critical thinking, supported by the literature. Nevertheless, the lack of linearity in the application of these strategies is also common. These are not enunciated in the curricula or in the teaching plans, at least with sufficient clarity to determine that, effectively, the teaching activity is being guided toward acquiring the R&CT capacity. The principal teaching and learning strategies made explicit in the curricula were: Portfolio of evidence, analysis of lectures, group dynamics, individual and group practices, research, problem-based learning (PBL), brainstorming, analysis and synthesis, conceptual maps, analogies, case studies, experiential workshops, nursing process, panel, forums**,** seminars, reflective diary, critical incident, essays, fieldwork, socio-drama, debate, dialogue, summaries, simulation laboratories, supervised clinical practice, among others.

### Evaluations forms suggested in the Nursing Curricula linked to achieving R&CT capacity

The evaluation activities identified in the curricula reviewed were: portfolio of evidence, field diaries or logbooks, knowledge test, practical exam, elaboration of research work, essays, as well as case studies, elaboration of the nursing process, clinical case, written exam, oral exam, and self-evaluation. Regarding the evaluation, it was found that know-how is mainly evaluated. There is a message/discourse on constructivism, capacities, flexibility, self-learning, *etc*., which is not congruent with the didactic-pedagogic techniques of transmitting knowledge, generally evaluated to pass a course and promote students to the next academic level.

### Coherence between R&CT capacity and educational methodology used in teaching, learning, and assessment processes

The teaching and learning strategies enunciated in the curricula reviewed are diverse. It is common to denominate active, creative, reflective, and critical or innovative methodologies. Most of the teaching plans gather the emerging tendencies having to do with learning and evaluation activities, although full coherence is not observed in them among the learning results expected, the teaching-learning activities developed and those activities to be evaluated by said results. Most of the curricula reviewed in this study do not make explicit the linearity between skill and the teaching-learning-assessment activities (T-L-E).

## Discussion

The R&CT capacity is currently a necessary capacity highlighted, explicitly and narratively, in all the Nursing Curricula reviewed in this research; although it must be specified that they must be expressed through diverse nominations, which permits concluding that the term can be seen as multi-voiced.(9) Likewise, shared interest exists in the curricula on the development and promotion of R&CT in the students, which favors providing formal or therapeutic care to users of nursing services. Big similarities are observed in the institutional perception on the need to develop the R&CT capacity in nursing students, linking it to clinical judgment, teaching, and research.(13) It has been verified that significant stress exists in the discourse and curricular organization. Incongruence is found and a clear inclination toward the formation of professionals with broad technical skills under a traditional, memory, banking and knowledge accumulation education.(14)

It is interesting to find in the graduation profile of the curricula analyzed statements that describe the formation of nursing professionals committed with the health of the population, development of their discipline, with capacity to construct their own knowledge, take initiative, and solve problems; this implies collecting, interpreting, evaluating, and selecting information for the purpose of making timely decisions (15) on the formation of competitive professionals and with aptitude for team work, assuming responsibly the risks implied by the interdisciplinary professional practice, capable of applying knowledge based on scientific evidence to care for human health with humanistic and bioethical sense, among other indications; however, the contents of the learning units, orientation of the curriculum, the methodology and teaching and learning strategies do not reflect clearly and congruently the achievement of these cognitive, attitudinal, ethical, and humanist skills founded and supported by the development of R&CT.

The social, cultural, health, and educational needs, to mention some, of the person, family, and society together are based on all the curricula reviewed, an aspect not reflected in the internal congruency of the study plan; particularly, in the profession’s object of study, its objectives, and the graduate’s profile, still privileging a biological, ahistorical, and individual education, although enunciating an integral or holistic formation of the graduate.(16) We believe there is stress or partial incongruence between that consigned in each of the parts that comprise the formal curriculum with the graduate’s profile.

Moreover, the gap is often observed between the curricular theory and the pedagogical practice consigned in the majority of the curricula studied. Adaptation of the traditional nursing curriculum to a skills-based curriculum has assumed an important advance for the nursing formation. In the first place, it has mean tan effort of reflection shared by the faculty to become conscious of the new approaches the nursing career must assume, in order to reduce the gap between theory and practice. Integration of contents, coordination among faculty staff, and transversality, as well the orientation toward the professional practice, have been elements present in the reflection carried out. Change is evident in the elaboration of the curriculum, above all in that referring to the orientation by skills and to more creative and innovative teacher methodologies. However, these changes are only evident at explicit curriculum level. When delving into the study of the curriculum, many questions remain unsolved, especially regarding the implicit or hidden curriculum. This poses the question of whether the nursing teachers in the study field are actually carrying out the pedagogical and didactic practices that the new curricular approach suggests in the academic program.(6)

With respect to the teaching, learning, and evaluation strategies, it may be said that these are not only multiple and diverse, but that they do not present full congruence, such is the case, for example, of the “teaching and learning strategy” used by the evidence portfolio and where the “evaluation” section specifies that the activity and the assignment will be graded through a written test of knowledge. We deem it important to reiterate that, during the second decade of the 21^st^ century, formation of nursing professionals must respond in efficient, integral, timely, assertive, and humanistic manner to the demands required by caring for life and maintenance and reestablishment of human health. This is not an easy task, given that the formal care provided by this professional implies the dynamic of growing changes in current university designs and methodologies claim with it implementation of an integrating version and transversal of the contents to deploy effectively the necessary elements to achieve strengthening the skills a nursing degree graduate must have.

Although it is true that the curriculum orientation is perceived inserted onto the constructivist current, prevalence is still noted in the curricula of technical-instrumental formation characteristic of the positivist biomedical paradigm, known for high percentages of contents with disease-focused biologic clinical character in detriment of contents oriented at health and social critique. The paradigm of the transformation in which we are immersed and which promotes the emancipation of the professionals, is scarcely supported on the formation of new professionals, which proceed and continue to form in a context marked by the repetitive and anachronistic practice of the traditional health culture, where medical paternalism continues expressing the power they maintain in healthcare structures. Nevertheless, it is necessary to highlight the effort by nursing education institutions to adjust the curricula to this new transforming paradigm, in spite of the cultural barriers instituted and which, in many cases is maintained by the care professionals themselves.

It is prudent to remember and recognize that the evaluation should be diagnostic, formative and summative or final, which seeks to use instruments that enable feedback from the educational process, preserving respect for the academic freedom of teachers, by these being autonomous institutions, through traditional evaluation techniques or assignment of research work, essays, case study, elaboration of the nursing process, clinical case, oral exam, self-evaluation, among other forms and strategies, which will grade the domain of *knowing, know-how, and knowing-being,* as general consideration of the curriculum, and *knowing to coexist* for nursing.(9) In spite of the aforementioned, 62% of the curricula analyzed, privilege the traditional evaluation, fundamentally written, which evidences the *objectification* only of knowledge, many times of memory type, where we believe that the principal results are limitation of the students’ creativity, autonomy, and self-criticism to measure and assess their academic performance and achievement.

## Conclusions

Development and promotion of reflective and critical thinking in nursing professionals is currently essential, given that the epistemological and sociological growth of the nursing profession require professionals with permanent discernment, supported on scientific, ethical, aesthetic, and personal knowledge. Reflection and critique, as learning tools, must be incorporated into the formation of nursing professionals. If learning based on life experiences is so important in nursing, reflection is vital to avoid repeating practices that hinder professional development and affect the quality of service provided by the nursing staff. This type of multi-referred thinking must be one of the essential axes that transform the professional formation and practice and with it the quality of care to users, family, and society, besides achieving higher professional status in nursing.

The results previously indicated permit stating that it is fitting to review and reorient institutional development plans, programs and academic plans, or study plans, fundamentally in what refers to the curricular contents, to develop and promote in the students and future graduates the practice of R&CT, diminishing existing gaps among theoretical formation, professional practice, and care requirements, reestablishing and maintaining health, favoring individual and collective wellbeing indices, especially if the aspiration of public and private universities that offer the nursing degree in Iberian America, consists in forming and contributing an interpretive vision to nursing professionals, through an academic curriculum that goes beyond teaching the diverse functions -granting, education, research, management, and administration, among others, which will take place around formal or professional nursing care, developing innovative skills. One of the cross-sectional-type curricular axes that must be considered, based on our point of view, is development, implementation, and promotion of R&CT in academic programs and their respective assignments or learning units, which provide nursing professionals the theoretical-practical tools necessary to use this type of thinking in any setting of the labor market they enter, which will enhance their vision, leadership, decision making, autonomy, and social and professional recognition.

The principal limitation noted in a multicenter research of this nature was the collection of information in the different participating countries, to the extent in which, although a document was constructed to obtain data, the interpretations made difficult some aspects of analysis and construction of analysis categories.
